# Novel homozygous pathogenic mitochondrial *DNAJC19* variant in a patient with dilated cardiomyopathy and global developmental delay

**DOI:** 10.1002/mgg3.1969

**Published:** 2022-05-25

**Authors:** Abeer Al Tuwaijri, Yusra Alyafee, Mashael Alharbi, Maryam Ballow, Mohammed Aldrees, Qamre Alam, Rola A. Sleiman, Muhammad Umair, Majid Alfadhel

**Affiliations:** ^1^ Medical Genomics Research Department, King Abdullah International Medical Research Center (KAIMRC), King Saud Bin Abdulaziz University for Health Sciences, King AbdulAziz Medical City Ministry of National Guard Health Affairs Riyadh Kingdom of Saudi Arabia; ^2^ Dr. Sulaiman Al‐Habib Group Al‐Rayan Hospital Riyadh Saudi Arabia; ^3^ Genetics and Precision Medicine Department (GPM), King Abdullah Specialized Children's Hospital King Saud Bin Abdulaziz University for Health Sciences, King Abdulaziz Medical City, Ministry of National Guard Health Affairs (MNG‐HA) Riyadh Saudi Arabia

**Keywords:** 3‐methylglutaconic aciduria, cardiolipin, dilated cardiomyopathy, *DNAJC19*, global developmental delay, homozygous, mitochondria

## Abstract

**Background:**

Dilated cardiomyopathy with ataxia syndrome (DCMA) or 3‐methylglutaconic aciduria type V is a rare global autosomal recessive mitochondrial syndrome that is clinically and genetically heterogeneous. It is characterized by early‐onset dilated cardiomyopathy and increased urinary excretion of 3‐methylglutaconic acid. As a result, some patients die due to cardiac failure, while others manifest with growth retardation, microcytic anemia, mild ataxia, and mild muscle weakness. DCMA is caused by variants in the DnaJ heat shock protein family (Hsp40) member C19 gene (*DNAJC19*), which plays an important role in mitochondrial protein import machinery in the inner mitochondrial membrane.

**Methods:**

We describe a single affected family member who presented with cardiomyopathy, global developmental delay, chest infection, seizures, elevated excretion of 3‐methylglutaconic acid, and 3‐methylglutaric acid in the urine.

**Results:**

Whole‐exome sequencing followed by Sanger sequencing revealed a homozygous frameshift variant in the reading frame starting at codon 54 in exon 4 in the *DNAJC19* gene (c.159del [Phe54Leufs*5]), which results in a stop codon four positions downstream. Quantitative gene expression analysis revealed that *DNAJC19* mRNA expression in this patient was substantially reduced compared to the control.

**Conclusions:**

We present a novel variant in the *DNAJC19* gene that causes rare autosomal recessive mitochondrial 3‐methylglutaconic aciduria type V. By comparing the current case with previously reported ones, we conclude that the disease is extremely heterogeneous for reasons that are still unknown.

## Graphical abstract

Using WES followed by Sanger sequencing we identified a novel homozygous variant in the exon 4 of the DNAJC19 gene (c.159del [Phe54Leufs*5]). Quantitative gene expression analysis showed that the DNAJC19 mRNA expression in our patient was substantially reduced compared to the control. The present study represents the first report of a DCMA syndrome in the Middle East.

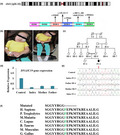

## 1 INTRODUCTION

Inborn errors of metabolism (IEM) are a large heterogeneous group of diseases that result from spontaneous or inherited mutations that affect enzyme activity or protein transport and lead to defects in metabolic pathways (El‐Hattab & Scaglia, 2016). The majority of these disorders manifest during childhood, and because of their heterogeneity, different IEM diseases have distinct epidemiologies, presentations, and heritabilities (Jones et al., 2021). Cardiomyopathies are a heterogeneous group of myocardial diseases with a wide range of causes, including generalized inherited mitochondrial disorders, which have an estimated prevalence of just over one in 5000 births (Meyers et al., 2013). Mitochondrial dysfunction is involved in the pathogenesis of multiple diseases, including dilated cardiomyopathy with ataxia syndrome (DCMA) or 3‐methylglutaconic aciduria type V syndrome (3MGA type V) (OMIM 610198) (Ucar et al., 2017). It was first characterized among the Canadian Dariusleut Hutterite population, which has the largest number of affected people globally (Davey et al., 2006).

3MGA type V is a rare autosomal recessive multiorgan disorder that manifests in the early infantile period. It has a variable phenotype that includes severe, early‐onset dilated cardiomyopathy; long QT syndrome is frequently observed. Mild to borderline non‐progressive developmental delay, growth failure, including significant motor delays, normochromic microcytic anemia, and male genital anomalies can also occur (Bertero et al., 2020). In addition, several patients have a significant increase in biochemical markers of mitochondrial dysfunction in the plasma and urine, particularly 3‐methylglutaconic acid (3‐MGC) and 3‐methylglutaric acid (3‐MGA) (Bertero et al., 2020). 3MGA type V is caused by mutations in the DnaJ heat shock protein family (Hsp40) member C19 (*DNAJC19*) gene, located on chromosome 3q26 (Sparkes et al., 2007). Although, the disease mechanism of the 3MGA type V phenotype is well studied, the reasons for its heterogeneity between patients remain elusive. Recent research has begun to shed light on the cellular phenotypes of DCMA and the function of the DNAJC19 protein. DNAJC19 is homologous to yeast that shares sequence similarities with the known Pam18/Tim14 (yPam18) protein (Richter‐Dennerlein et al., 2014). This protein regulates cardiolipin remodeling by interacting with protein complexes known as prohibitins that form protein scaffolds and lipids in the inner membrane of mitochondria, which is essential for mitochondrial morphogenesis and metabolism (Richter‐Dennerlein et al., 2014).

**FIGURE 1:**
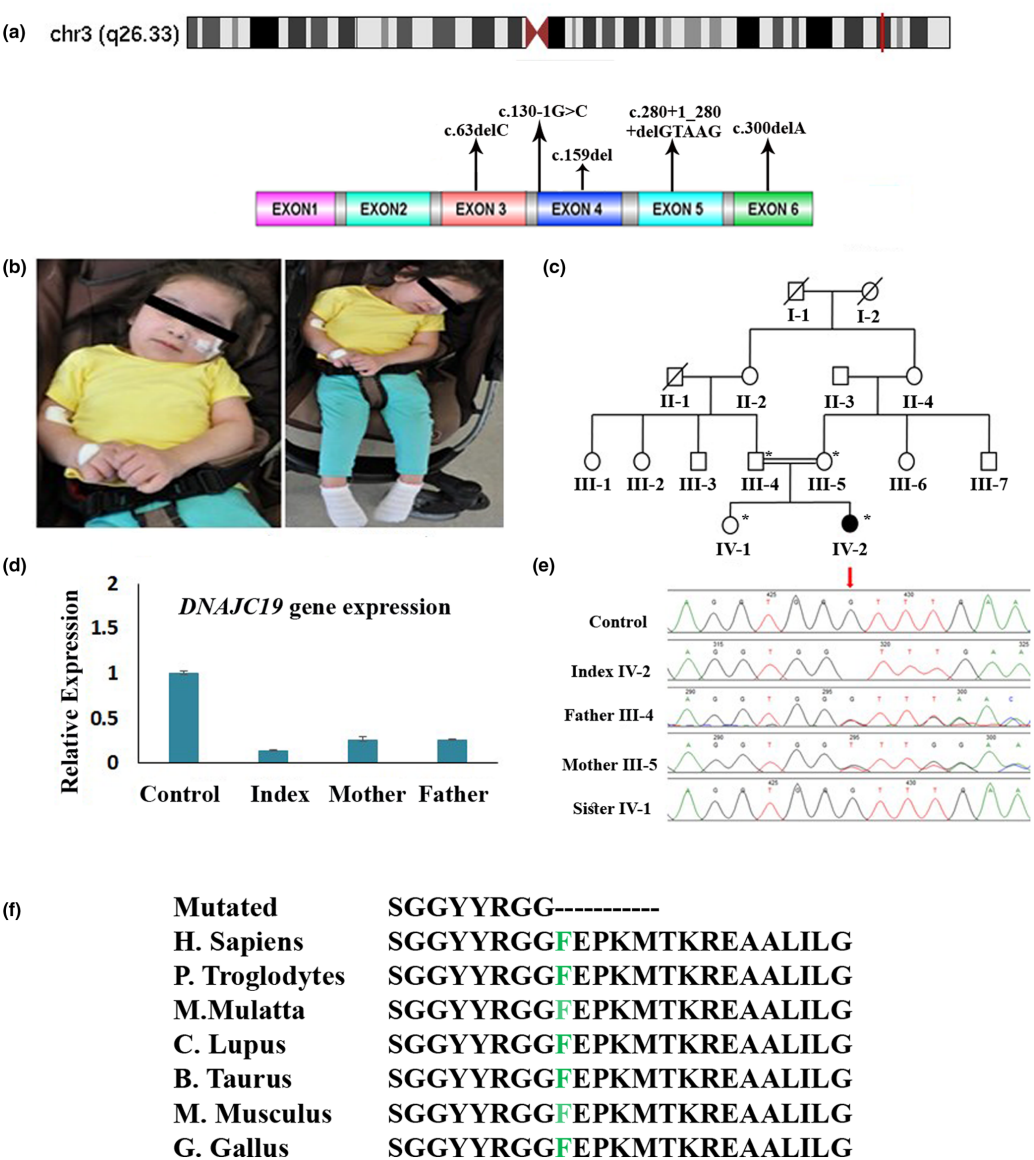
(a) Schematic diagram of the *DNAJC19* gene showing the patient's variant located in exon 4 in chromosome 3 and location of the previously reported pathogenic variants. (b) Photographs showing the phenotypes of the patient. Written informed consent was obtained from the parents to publish images. (c) Pedigree of the family with consanguineous marriage with an autosomal recessive inheritance pattern. The filled symbol represents affected, while the unfilled one represents unaffected individuals. Double lines indicate a consanguineous marriage. A cross line on the symbol indicates a deceased individual. (d) Relative mRNA expression levels of *DNAJC19* gene of the affected patient compared to the parents (carriers) and control. The results showed the *DNAJC19* gene expression is significantly decreased in the affected patient compared to control. (e) DNA sequence chromatograms showing the variants in the DNAJC19 gene in the homozygous affected, heterozygous, wild type sister, and a control sample. (f) Partial sequence of the DNAJC19 amino acids, showing the conservation of phenylalanine amino acid across different species

3MGA type V shares some similarities with X‐linked Barth syndrome, caused by a genetic mutation in the tafazzin (*TAZ*) gene. These include dilated cardiomyopathy, increased 3‐methylglutaconic acid, and 3‐methylglutaric acid levels, and growth failure. The molecular pathogenesis in both syndromes leads to the disruption of the normal cardiolipin pool of the inner mitochondrial membrane (Ucar et al., 2017). The distinctive differences in the clinical presentations of these syndromes include the absence of skeletal myopathy and neutropenia in people with 3MGA type V (Rohani et al., 2020). According to the Human Gene Mutation Database (HGMD®), only six mutations causing 3MGA type V have been reported. Also, there are only 56 entries for the *DNAJC19* gene in ClinVar (Figure 1a).

In this study, we identified a novel homozygous *DNAJC19* gene variant in a 4‐year‐old Arabic female patient with cardiomyopathy and 3MGA type V. This is the first report of DCMA syndrome in the Middle East.

## 2 MATERIALS AND METHODS

### 2.1 Genomic DNA extraction

Peripheral blood samples from the patient and her family were collected in EDTA tubes. Genomic DNA was extracted from whole blood using a QIAamp Blood Midi Kit (Qiagen, Germantown, MD, USA). DNA concentration was quantified using the NanoDrop‐1000 spectrophotometer.

### 2.2 Whole‐exome sequencing (WES)

Genomic DNA was sequenced using the Ion Torrent (Ion S5 XL, Thermo Fisher Scientific, Waltham, MA, USA) platform data from the S5 XL. Runs were initially processed using Torrent Suite v4.0 (Thermo Fisher Scientific) to generate sequence reads, trim adapter sequences, filter, and remove poor signal profile reads. All the reads were aligned against human assembly hg19 (GRCh37; http://genome.ucsc.edu/). Variant calling from the Ion AmpliSeq (Thermo Fisher Scientific) sequencing data were generated using the Torrent Variant Caller (TVC) to eliminate incorrect base calling. Three filtering steps were used to generate the final variant calling file (VCF) (Alfadhel et al., 2021; Umair et al., 2021).

### 2.3 Bioinformatics analysis

The VCF was analyzed using BaseSpace (Illumina, San Diego, CA, USA) software for variant interpretation. All possible diseases caused by variants reported in the HGMD®, ClinVar, CentoMD, and PubMed databases, including all variants with minor allele frequencies of less than 1% in ExAC (http://exac.broadinstitute.org/), gnomAD (https://gnomad.broadinstitute.org/), and 1000 Genomes (http://www.internationalgenome.org/), were considered. All identified variants were evaluated with respect to their pathogenicity and causality. Variant classification based on the recommendations of the American College of Medical Genetics and Genomics was followed.

### 2.4 Sanger sequencing validation and co‐segregation analysis

Sanger sequencing was applied to confirm the identified variant in all family members. The variant (c.159del [Phe54Leufs*5]) in the *DNAJC19* gene (NM_145261.3) was amplified and analyzed by PCR using the standard method. The Primer3 online tool (http://bioinfo.ut.ee/primer3‐0.4.0/) was used to design forward (5′‐TCCTTTA CTCCCGGAAGACCT‐3′) and reverse (5′ACAAAATCCC CCATGACCCTC‐3′) primers. The product size was 479 bp and the annealing temperature was (60°C). The product was sequenced by Sanger sequencing using capillary electrophoresis through ABI 3730xl (Alfadhel et al., 2019).

### 2.5 RNA extraction and quantitative real‐time PCR

To investigate the effect of the identified variant on *DNAJC19* gene expression, RNA was extracted from peripheral blood mononuclear cell samples (PBMCs) of the patient and her family (mother, father, and sister), as well as the control, using a standard TRIzol RNA isolation protocol (Invitrogen, Carlsbad, CA, USA). RNA quantification and purity were tested using standard methods. The obtained RNA was then reverse‐transcribed using a high‐capacity cDNA reverse transcription kit (Applied Biosystems/Thermo Fisher Scientific). The qPCR reaction was conducted using a SYBR Green Master Mix (Applied Biosystems/Thermo Fisher Scientific) on a QuantStudio 6 Flex Real‐Time PCR System (Applied Biosystems/Thermo Fisher Scientific). The primer sequences for the *DNAJC19* cDNA can be provided upon request. All tested samples were normalized to the *GAPDH* gene as an endogenous control, and all reactions were repeated independently and performed in three replicate experiments (Asiri et al., 2020).

### 2.6 Statistical analysis

The data for the quantitative real‐time PCR results were analyzed using one‐way analysis of variance (one‐way ANOVA). A *p*‐value <0.05 was considered statistically significant. All calculations were performed using the Statistical Package for the Social Sciences (SPSS) version 26.0 (IBM Corp., Armonk, NY, USA).

## 3 RESULTS

### 3.1 Case presentation

The patient is currently 4 years and 10 months old and has a rare combination of cardiomyopathy and global developmental delay (Figure 1b). She is the younger of two female children of the first cousin's parents (Figure 1c). She was born at full term via normal spontaneous vaginal delivery, weighing 2.7 kg (5th–10th percentile), measuring 46 cm (5th–10th percentile), and having a head circumference of 32 cm (3rd percentile). She was discharged along with her mother, who was in good health. The patient's first cause for concern was a febrile illness at 14 months. During the routine examination, the doctor noticed a cardiac murmur. The patient's condition deteriorated, and she developed a chest infection. She was admitted to the hospital, and cardiomyopathy was detected, which was initially attributed to the viral infection. Following discharge, it was discovered that the patient had a developmental delay. She was unable to walk or speak and had tremors in her hands, so a magnetic resonance imaging (MRI) scan and electroencephalogram (EEG) were performed; they were both normal.

With time, the patient gained some developmental skills, such as walking with assistance and speaking around 20–30 single words. Following that, at the age of 3 years and 4 months, she collapsed and was tonic in posture while going about her daily routine. When her parents brought her to the hospital, it was discovered that she was in cardiac arrest. She was resuscitated before being admitted to the pediatric intensive care unit for 3 weeks. The brain MRI revealed extensive high T2 signal intensity and restricted diffusion in both cerebral hemispheres. Biochemical analyses of organic acids were performed using blood and urine samples. The patient's 3‐methylglutaconic acid levels were significantly elevated in addition to fumaric acid, glutaric acid, and 3‐methylglutaric acid; there were also traces of 3‐hydroxybutyric acid (Table 1). She was referred to a genetics clinic for further evaluation at the age of 3 years and 10 months, and her examination revealed the following growth parameters: weight: 10.7 kg (<3rd percentile), height: 89 cm (<3rd percentile), and head circumference: 47 cm (3rd–5th percentile). There were no apparent dysmorphic features. Central hypotonia and spastic quadriplegia were discovered during a neurological examination. An S1 + S2 + systolic murmur grade 3/6 in the left lower costal margin was discovered during her cardiovascular examination. The results of the other systemic examinations were unremarkable.

**TABLE 1:** Biochemical tests and organic acid screen

Amino acids	Result	Unit	Reference range
Phosphoserine	13	μmol/L	1–30
Taurine	55	μmol/L	10–170
Phosphoethanolamine	53	μmol/L	<69
Aspartic acid	8	μmol/L	1–24
Hydroxyproline	17	μmol/L	3–45
Threonine	79	μmol/L	35–226
Serine	106	μmol/L	69–187
Asparagine	81	μmol/L	23–112
Glutamic acid	55	μmol/L	5–150
Glutamine	341	μmol/L	254–823
Sarcosine	0	μmol/L	<9
Proline	90	μmol/L	59–369
Glycine	152	μmol/L	127–341
Alanine	304	μmol/L	152–547
Citrulline	8	μmol/L	1–46
Alpha aminobutyric acid	30	μmol/L	4–31
Valine	236	μmol/L	74–321
Cystine	↓ **3**	μmol/L	5–45
Methionine	21	μmol/L	7–47
Cystathionine	0	μmol/L	<3
Isoleucine	83	μmol/L	22–107
Leucine	160	μmol/L	49–216
Tyrosine	42	μmol/L	24–115
Phenylalanine	77	μmol/L	26–91
Beta alanine	0	μmol/L	<7
Tryptophan	20	μmol/L	<79
Lysine	103	μmol/L	48–284
3‐Methylhistidine	↑ **5**	μmol/L	<5
Histidine	79	μmol/L	41–125
Arginine	20	μmol/L	10–140

Organic acid	
3‐methylglutaconic acid	Increased
3‐methlglutaric acid	Increased
Fumaric acid	Increased
Glutaric acid	Increased
3‐hydroxybutyric acid	Increased

She is now 4 years and 10 months old, and she continues to have seizures in the form of blinking of the eyes several times throughout the day. The seizures are managed with levetiracetam and valproic acid. She also has a global developmental delay; she can sit but cannot walk and is confined to a wheelchair. Due to her severe complications, she only uses single words to express her needs and is not enrolled in school or any academic rehabilitation centers. Her cardiac examination remains stable, with an echocardiogram revealing a 57 percent ejection fraction.

### 3.2 WES identified a novel variant in the *DNAJC19* gene

WES was performed on the DNA of the affected child and her family using the Ion Torrent (Ion S5 XL) platform with an average coverage depth of 20× for >98% of the targeted bases. Data analysis revealed the presence of a single homozygous frameshift site starting at codon 54 in exon 4 in the *DNAJC19* gene (c.159del [Phe54Leufs*5]) (NM_145261.3).

### 3.3 Sanger sequencing

The frameshift variant identified by WES was confirmed with Sanger sequencing and was found to segregate with the disease phenotype (Figure 1e). The patient is homozygous, while her parents are heterozygous (c.159del [Phe54Leufs*5]). This variant has never been reported in public databases, including dbSNP, 1000 Genomes, internal database (in‐house), and ExAC. This variant was investigated and found to be absent in the Saudi genome project from unrelated Saudi Arabian individuals, which eliminates the possibility that the variant we found is a single nucleotide polymorphism and is giving redundant risk information. The identified variant c.159del creates a shift in the reading frame starting at codon 54. The new reading frame ends in a stop codon four positions downstream. There is a phenotypic overlap of the patient with the indicated frameshift variant in the *DNAJC19* gene. However, the loss of function mechanism has not yet been clearly described for this gene. Therefore, according to the American College of Medical Genetics and Genomics recommendations, it is classified as a variant of uncertain significance (class 3).

### 3.4 Quantitative PCR

Using RT‐qPCR, *DNAJC19* mRNA expression in the PBMCs was assessed in the affected patient, parents, and control individuals. The RT‐qPCR data revealed that the affected patient with the homozygous variant (c.159del) had a substantial reduction in the relative gene expression of the *DNAJC19* gene compared to the parents and control (Figure 1d).

## 4 DISCUSSION

An increasing number of IEMs that affect mitochondrial energy metabolism and are associated with abnormal phenotypic feature, such as elevated urinary 3‐methylglutaconic acid excretion (Machiraju et al., 2021). A biochemical finding is commonly found in a heterogeneous group of metabolic diseases, especially mitochondrial disorders. This includes 3‐methylglutaconyl‐CoA hydratase deficiency (3MGA type I, 3MGA1) (OMIM #250950), Barth syndrome (3MGA type II, 3MGA2) (OMIM #302060), Costeff optic atrophy syndrome (3MGA type III, 3MGA3) (OMIM #258501), 3MGA type IV (3MGA4) (OMIM #250951), and 3MGA type V (3MGA5) (OMIM #610198). The (I–V) Roman numerical order is according to the order of their discovery, irrespective of their pathomechanism (Wortmann et al., 2013). The 3MGA type I phenotypes include a decreased activity of 3‐methylglutaconyl‐CoA hydratase, which has a significant role in converting 3‐methylglutaconyl‐CoA to 3‐hydroxy‐3‐methylglutaryl‐CoA. Both 3MGA type II and 3GMA type V are caused by abnormal inner mitochondrial membrane proteins (Machiraju et al., 2021). 3MGA type III, also known as optic atrophy plus syndrome, is caused by a defect in a protein with an uncharacterized function located in the outer mitochondrial membrane. 3MGA type IV represents an unclassified heterogeneous group of individuals who share a continuous or intermittent increase in urinary 3‐methylglutaconic acid excretion (Wortmann et al., 2013).

The outcome of this era of molecular genetic tests is providing a huge leap forward in the diagnosis of pediatric patients with multiple organ diseases (Frésard & Montgomery, 2018). Genetic testing, including WES, is becoming much more common in clinical practice because it provides an unbiased analysis of all protein‐coding sequences (Frésard & Montgomery, 2018). In this article, we report a female patient with dilated cardiomyopathy and global developmental delay, which suggests the importance of performing metabolic screening. In addition, we found a significant increase in 3‐methylglutaconic aciduria in our patient. These phenotypes are shared between different well‐characterized diagnoses, including 3MGA type V syndrome, caused by a mutation in the *DNAJC19* gene. They include Sengers syndrome (caused by a mutation in the *AGK* gene) and Barth syndrome (caused by a mutation in the *TAZ* gene) (Wasmus & Dudek, 2020). The latter two were excluded because of the absence of skeletal myopathy; furthermore, Barth syndrome is an X‐linked syndrome, and our patient is female (Wasmus & Dudek, 2020).

After conducting WES and confirming the result with Sanger sequencing, we discovered that our patient carries a homozygous variant of uncertain significance (c.159del) identified in the *DNAJC19* gene located on chromosome 3. This region is highly conserved across multiple species (Figure 1f). Moreover, the RT‐qPCR data showed a significant decrease in mRNA expression in our patient sample with the mutant *DNAJC19* compared to her family members and the control sample. It might be possible that the heterozygous mutation present in the carrier individuals is causing a decreased transcription of the *DNAJC19*, however, the protein expression might be sufficient for the physiological activity.

By comparing the current case with previously reported cases in the literature, the common phenotypic characteristics of 3MGA type V syndrome that have presented in the majority of cases are increased 3‐methylglutaconic aciduria (100%) and growth failure (100%), followed by developmental delay (77.5%), dilated cardiomyopathy, and microcytic hypochromic anemia presented in (63%) and (63.6%), respectively (Table 2) (Al Teneiji et al., 2016; Ojala et al., 2012). The index (IV‐2) did not have dysmorphic features, microcytic anemia, or hepatic dysfunction, although these phenotypes have been observed in several cases with 3MGA type V. The *DNAJC19* gene is localized to the inner mitochondrial membrane (Ojala et al., 2012). Richter‐Dennerlein et al. (2014) showed that the DNAJC19 protein interacts and creates a functional complex with prohibitions. Microscopic examination of heat tissue obtained from a deceased 3MGA type V child showed subendocardial fibrosis and prominent perinuclear vacuolation of cardiomyocytes, and increased numbers of mitochondria with scattered electron‐dense inclusions (Machiraju et al., 2021). In addition, live tissue exhibits severe macro vesicular steatosis with clear vacuoles existing through all liver cells (Machiraju et al., 2021).

**TABLE 2:** Summary of phenotypic characteristics of cases with 3‐methylglutaconic aciduria, type V syndrome compared to the current case

	Davey et al. (2006) (*n* = 18) [5]	Ojala et al. (2012) (*n* = 2) [14]	Al Teneiji et al. (2016) (*n* = 1) [15]	Ucar et al. (2017) (*n* = 1) [4]	Machiraju et al. (2021) (*n* = 43) [10]	Our patient (*n* = 1)	Total
Ancestry	Canadian Hutterite	Finnish	Not reported	Turkish	Canadian Hutterite	Arab	
Gender/age mean	11 male, 7 female (11 alive), (7 deceased)/mean age 16 month	2 male (1 alive), (1 deceased)/mean age 15 month	1 alive/13 years	1 deceased/3 years	24 male, 19 female	1 alive/4 years	
cDNA change	Homozygous c.130‐1G>C Exon 4	Homozygous c.300delA, (p. Ala101Profs*10) Exon 6	Homozygous c.280+1_280+5delGTAAG Exon 5	Homozygous c.63delC, (p.Tyr21*) Exon 3	Homozygous c.130‐1G>C Exon 4	Homozygous c.159del (p.Phe54Leufs*5) Exon 4	
Mutation type	Splice site	Frame shift	Splice site deletion	Stop mutation	Splice site	Frame shift	
Consanguinity	No	No	Yes	Yes	No	Yes	
IUGR	8:18	0:2	Not reported	1:1	Not reported	0:1	9/22 (41%)
Growth failure	18:18	2:2	1:1	1:1	Not reported	1:1	23/23 (100%)
Dysmorphic features	0:18	0:2	0:1	1:1	Not reported	0:1	1/23 (4.3%)
Dilated cardiomyopathy	12:18	2:2	1:1	1:1	9:18	1:1	26/41 (63%)
Prolonged QT interval	6:18	2:2	1:1	Not reported	15:18	1:1	25/40 (62.5%)
Developmental delay	10:18	Not reported	1:1	1:1	18:19	1:1	31/40 (77.5%)
Abnormal vision	4:18	0:2	Not reported	0:1	10:11	1:1	15/33 (45%)
Seizure	2:18	0:2	0:1	0:1	5:20	1:1	8/42 (17%)
Microcytic hypochromic anemia	12:18	2:2	Not reported	1:1	Not reported	0:1	14/22 (63.6%)
Hepatic dysfunction	8:18	1:2	1:1	1:1	Yes (the number was not indicated)	0:1	11/23 (47.8%)
Increase 3‐methylglutaconic aciduria	18:18	2:2	1:1	1:1	43:43	1:1	66/66 (100%)

To the best of our knowledge, this is the first case of a *DNAJC19* gene mutation reported in the Middle East with a distinctive phenotype characterized by early‐onset cardiomyopathy accompanied by global developmental delay. Enrolling more patients and performing advanced functional studies are essential for better insight into the molecular mechanism associated with 3MGA type V syndrome, which might uncover the crucial role of mitochondrial imports and trafficking players in human cardiomyopathies.

## AUTHOR CONTRIBUTIONS

Abeer Al Tuwaijri wrote the manuscript and performed analysis for Sanger sequencing and qPCR. Yusra Alyafee, Muhammad Umair, and Majid Alfadhel, analyzed the data, reviewed, and edited the manuscript. Majid Alfadhel responsible about patient clinical assessment and work supervision. Rola A. Sleiman, Maryam Ballow, Qamre Alam, Mohammed Aldrees, and Mashael Alharbi, performed the experimental works.

## CONFLICT OF INTEREST

The authors have no conflict of interest to declare.

## ETHICAL COMPLIANCE

This research study was approved by the Institutional Review Board of King Abdullah International Medical Research Center (KAIMRC), Riyadh, Saudi Arabia. The patient was fully examined at the Genetics clinic of King Abdulaziz Medical City in Riyadh, Saudi Arabia. Written informed consent to perform clinical genomic analysis and data publication was obtained and signed from the parents.

## Data Availability

The data that support the findings of this study are available from the corresponding author upon reasonable request.
